# Informal support to first-parents after childbirth: a qualitative study in low-income suburbs of Dar es Salaam, Tanzania

**DOI:** 10.1186/1471-2393-11-98

**Published:** 2011-11-29

**Authors:** Columba K Mbekenga, Andrea B Pembe, Kyllike Christensson, Elisabeth Darj, Pia Olsson

**Affiliations:** 1 Department of Community Health Nursing, School of Nursing, Muhimbili University of Health and Allied Sciences, Dar es Salaam, Tanzania; 2 Department of Women's and Children's Health, Uppsala University, Uppsala, Sweden; 3 Department of Obstetrics and Gynaecology, School of Medicine, Muhimbili University of Health and Allied Sciences, Dar es Salaam, Tanzania; 4 Department of Women's and Children's Health, Karolinska Institute, Stockholm, Sweden

## Abstract

**Background:**

In Tanzania, and many sub-Saharan African countries, postpartum health programs have received less attention compared to other maternity care programs and therefore new parents rely on informal support. Knowledge on how informal support is understood by its stakeholders to be able to improve the health in families after childbirth is required. This study aimed to explore discourses on health related informal support to first-time parents after childbirth in low-income suburbs of Dar es Salaam, Tanzania.

**Methods:**

Thirteen focus group discussions with first-time parents and female and male informal supporters were analysed by discourse analysis.

**Results:**

The dominant discourse was that after childbirth a first time mother needed and should be provided with support for care of the infant, herself and the household work by the maternal or paternal mother or other close and extended family members. In their absence, neighbours and friends were described as reconstructing informal support. Informal support was provided conditionally, where poor socio-economic status and non-adherence to social norms risked poor support. Support to new fathers was constructed as less prominent, provided mainly by older men and focused on economy and sexual matters. The discourse conveyed stereotypic gender roles with women described as family caretakers and men as final decision-makers and financial providers. The informal supporters regulated the first-time parents' contacts with other sources of support.

**Conclusions:**

Strong and authoritative informal support networks appear to persist. However, poverty and non-adherence to social norms was understood as resulting in less support. Family health in this context would be improved by capitalising on existing informal support networks while discouraging norms promoting harmful practices and attending to the poorest. Upholding stereotypic notions of femininity and masculinity implies great burden of care for the women and delimited male involvement. Men's involvement in reproductive and child health programmes has the potential for improving family health after childbirth. The discourses conveyed contradicting messages that may be a source of worry and confusion for the new parents. Recognition, respect and raising awareness for different social actors' competencies and limitations can potentially create a health-promoting environment among families after childbirth.

## Background

Maternal and infant mortality and morbidity during the period after childbirth is still high in many low-income countries, despite being prioritised in the millennium development goals (MDG) 4 and 5 [1,2]. In many sub-Saharan African countries, postpartum health programs have received less attention than antenatal and intrapartum programs [2,3]. Thus, after childbirth, many parents rely on informal support for health related matters [4-6].

The period after childbirth is stressful for parents, posing new life challenges and expanded responsibilities in relation to family life and health [7-9]. Tanzanian mothers report depressive symptoms [10] and concerns over tiredness, fatigue, infant crying, combining breastfeeding and increased workload [11-14], similar to reports from postpartum mothers in Western countries [7,15,16]. The mothers are worried over their partner relationship and are uncertain about the timing of sexual resumption and contraceptive use [11,12]. Tanzanian first time fathers describe challenges in balancing bread winning responsibilities, maternal and child care needs and sexuality after childbirth [17]. Both new mothers [12] and fathers [17] in low-income areas in Dar es Salaam express the need for more guidance on matters related to child and maternal care after childbirth. Insufficient support from the health care system and conflicting messages from different sources of support during postpartum are common [18]. This is also voiced by Tanzanian mothers [11-13] and fathers [17], despite the relative high presence of health care services in the studied area.

Social support enables a smooth transition to parenthood [5] and has a positive influence on health during the childbearing period [19]. Social support is defined as 'any emotional, informational, and tangible, and/or comparison, social resource provided to and perceived as effective by the recipient [19]. Moreover, social networks are regarded as an important aspect of 'social capital' that enables an individual to gain access to resources such as ideas, information, services and support that would otherwise be inaccessible [20]. High levels of social capital are associated with positive health effects [21], especially in low-income communities with poor structural facilities [22,23] where people may become more dependent on informal support networks. The extent of social support a person receives depends on the quality and quantity of social networks available to them [24]. In different settings, social support during the postpartum period has positive influence on breastfeeding practices [25-27] and mothers' depressive symptoms [4,28,29].

The migration of young rural population to urban city areas is increasing worldwide and about half the world's population is estimated to live in urban settings [30]. Internal migration is described as leading to social disruption [31,32], which renders migrant population more vulnerable, as they become disconnected from the social ties that provided social support. However, in low-income suburbs with multiple ethnic groups in Dar es Salaam, Tanzania, first-time parents are depending heavily on familial informal support for health related decision-making and care after childbirth [12,17] and both positive and negative health practices are encouraged by informal supporters. Thus, further knowledge on how informal support is understood by its stakeholders to be able to improve the health in families after childbirth is required. Such socially constructed meaning systems, or discourses, are inbuilt and taken-for-granted in ways people talk. This study aimed to explore discourses on health related informal support to first-time parents after childbirth in low-income suburbs of Dar es Salaam, Tanzania.

## Methods

### Study setting

The study was conducted in Ilala Municipal in the outskirts of Dar es Salaam, the largest city in Tanzania. Ilala has an estimated population of 637 000 [33] and most of its young inhabitants have migrated from rural areas in search of 'greener pastures' [34]. However, their expectations are seldom met, and they face high unemployment, congestion, and poor housing, factors that contribute to health risks [34,35].

In Ilala, one district hospital, two health centres, and 14 dispensaries provide reproductive and child health care (RCH) including antenatal care, delivery services, child health, family planning services, and voluntary counselling and testing for HIV. However, in the area, there are private and faith-based health facilities that contribute significantly to the provision of RCH care. Care during immediate postpartum is provided but there is no routine follow-up for mothers with uncomplicated births. In Dar es Salaam, about 90% of women give birth at health care facilities [36] and are usually discharged within 24 hours, if there are no complications. According to the Ministry of Health [37], postpartum follow-up of mothers should be provided in the RCH clinics at 7, 28 and 42 days after childbirth. However, this is seldom implemented and most mothers visit health clinics after 4-6 weeks for growth monitoring and immunisation of their infants [11]. Thus, most women and their families do not receive much support from the health care system during the postpartum period.

### Participants and recruitment

Purposive sampling [38] was used to capture variations in experiences of informal support after childbirth. The criteria for participation were first-time mothers and fathers who were living together with their partners and their infant aged 6 months or less, and women and men who had experience of supporting first-time parents. Local government authorities assisted the recruitment of participants who were recruited at street level after the researchers had explained the aim and procedures of the study, principles of voluntary participation and selection criteria. Subsequent participants were recruited by snowball sampling [38]: 82 participants (45 women, 37 men) from 29 different ethnic groups in Tanzania with an age range between 18-90 years were recruited. Most participants had 2-11 years of education; however, 10 participants had not received any formal education. The number of participants in each category and the focus group discussions (FGD) that were analyzed are summarized in Table 1.

**Table 1:** Participants and focus group discussions (FGD)

Participants	Age range (years)	Average age (years)	Number of participants	FGD conducted	FGD analysed
Mothers	18-30	21	24	4	3
Fathers	21-31	26	16	3	3
Female supporters	38-70	54	21	4	4
Male supporters	42-90	63	21	3	3

TOTAL			82	14	13

### Focus group discussions

Focus group discussion [39] was chosen for data collection as it captures group norms and meanings in the discussions between group participants. Fourteen FGD each with 4-8 participants were conducted between August 2009 and January 2010. The following piloted hypothetical scenario delineating various potential health problems after childbirth was read aloud to stimulate and guide the discussions:

*Neema is a young Chagga [ethnic origin] woman who is self-employed with a small hair salon in Buguruni. She is married to Peter and they were blessed with their first baby four months ago. Neema's husband, the young father Peter, is Hehe [ethnic origin] and a self-employed shopkeeper. He is very excited about their baby and feels guilty for not spending enough time with his new baby and partner. However, he cannot manage as his family depends on his daily earnings from his small shop*.

*As this was their first baby, they were not sure on how to take care of the cord and how Neema's hygiene and nutritional status should be maintained. Neema was also worried because she had vaginal discharge a few days after delivery. Neema's mother-in-law was applying hot water compressions but Neema complained that it was very hot and uncomfortable*.

*Neema is breastfeeding exclusively, as advised by the midwives. However, the baby has been crying, especially at night. They do not sleep much and feel stressed. Neema and Peter suspect that the baby is not getting enough breast milk and they are wondering whether they should start her with light porridge*.

*Peter wants them to resume sex, but Neema maintains they should abstain until the child has stopped breastfeeding i.e. one to two years. They had a big argument where Peter slapped her because she refused him sex. Neema worries that Peter might not abstain and hence become unfaithful*.

Thereafter, the discussion was opened by asking the participants how situations such as those depicted in the scenario could best be handled to promote family health in their context. Probing questions included would any kind of support be helpful, who would do what and why. Two of the authors (CKM, ABP) moderated the female and male groups respectively and ensured privacy during the discussions. The discussions were conducted in Swahili and audio-recorded. Field notes on non-verbal aspects and events happening during the discussion were taken.

### Discourse analysis

A discourse is a way of talking about or understanding the reality or particular aspects of the reality [40]. Discourse analysis is based on social constructionism and is a systematic analysis of different ways of talking about reality: the way people talk both reflects and transforms social practices [40]. Such socially constructed meaning systems, or discourses, are inbuilt and taken-for-granted in ways people talk. This analytical process was inspired by Parker [41] and began with reading the whole transcript to obtain an overview of how informal support was understood by the participants and what role it played among families after childbirth. Thereafter, all statements specifically referring to informal support in relation to family health were identified. This was followed by a circular process of detailed analysis, where the transcripts and statements were read, reflected over, and discussed between the authors and then organised into four main discourses.

### Ethical issues

Ethical clearance was approved by the Senate Research and Publication committee of Muhimbili University of Health and Allied Sciences. The Ilala Municipality Office granted permission to conduct this study. The Uppsala University Ethics Review Board conducted a consultative review. Verbal consent was obtained from each participant after receiving information on the purpose and procedure of the study and that participation was voluntary.

## Results

Four main discourses were identified in the FGD on informal support to first-time parents after childbirth (Table 2), which are presented with illustrative quotes from the FGD. The abbreviations P1, P2 etc are used to indicate different participants and M indicates the moderator of the FGD.

**Table 2:** Discourses on informal support after childbirth

1	Authoritative informal support maintained and reconstructed
2	Poverty and poor social relations restrict informal support
3	Informal support as gatekeeper to other forms of support
4	Upholding stereotypic norms of femininity and masculinity

### 1. Authoritative informal support maintained and reconstructed

The dominant discourse was that after childbirth a new mother needed and should be provided with support for care of the infant, herself and the household work by other women in the family. The main reason given for the support is the need for transferring knowledge and skills on the care of the infant and the mother. The new mothers' need for rest and recovery after childbirth was likewise described as important, although there were variations on the recommended duration.

P1: [the mother needs] training on how to take care of the child, that is to train her on how to breastfeed, to hold the child when breastfeeding (...)

P2: that is right, most often when a woman delivers, she is supposed to stay at her mother's or mother in law's home. It is not easy to go direct to your home [mmh]. (...). She needs help from her parents or from any person who is close to her.

M: Mmh! So, who exactly is supposed to provide that help?

P2: Mostly your parents or husband's parents [mothers].

P1: Mostly your mother-in-law or your mother or your sister-in-law or your sister.

FGD 3 Mothers

As seen in the quote above, maternal and paternal mothers or other close and extended family members were the key supporters and were portrayed as experts, spending much time with the new mothers and infants advising, role modelling and supervising on infant feeding and care, mother care and nutrition, sexuality, solving marital conflicts and helping with household chores. Thus, the informal support upheld part of the responsibilities of women in households that are important for maintaining and promoting health in families. Furthermore, identifying health problems in the family and explaining why they happened and what could be done to prevent and handle them, were described as part of the informal support. The new mother was expected to take over these responsibilities later when the informal supporters were gone. An integral component of the informal support was the transferral of social norms, and ensure adherence to these norms. The informal support was mainly depicted as much appreciated by new mothers.

There were four main constructs on how this support was organised. The main construct was that a female family member provided the support in the homes of the new parents, where she lived temporarily. In the absence of close or extended family members, neighbours and friends were depicted as taking the support role for the new family. This was similar to what is done in rural areas where relatives lived in the neighbourhood. Housemaids were mentioned as providing support when the family had enough money to employ one, which was seldom the case. To ensure optimal support, this meant the mother and the infant moved out of their home and stayed with their in-laws or the woman's parents for some weeks before and after childbirth.

The descriptions of support provided by the informal networks focused primarily on the mother and the infant and there was little attention to supporting the father. In the discourse, the new fathers' contribution to family health after childbirth was constructed as ensuring the mothers received nutritious food for early recovery and sufficient breast milk. Fathers were also expected to provide for the material needs of the infant, such as soap and nappies. The help with care and household chores by female supporters ensured the new fathers had time for paid employment, as they were typically considered the family's financial provider. New fathers were occasionally positioned as direct recipients of advice and support, mainly from older male relatives, in areas of economy and sexuality, which were depicted as a masculine concern for support. Paternal and maternal parents and other close family members were portrayed as possible supporters to new fathers through providing financial support and other baby/mother care materials that helped reduce household expenditure.

### 2. Poverty and poor social relations restrict informal support

Poverty was delineated as a main constraint for promoting health after childbirth. Provision of informal support was not straightforward and equally available for everybody. Social and economic status determined how much informal support new parents received. This was true for familial support but most obvious in the absence of family members and when a neighbourhood or friendship-based support was needed as demonstrated in the following quote:

I have seen people getting support, but nowadays, they look at people with money. You know, if you do not have money, they will look at you as if they don't see you and they would help a person with money (...) they ignore you as if you are no longer there, but if I have money [mmh] whatever I say, [I] get support.

FGD 11 Female supporters

The new fathers were expected to help in the household in case female informal supporters were unavailable. If the relatives were unable to provide this support, the new mother herself was expected to take on more responsibilities or arrange with female neighbours and friends to help.

The support received from family members, neighbours and other people was determined by how much the new parents, and especially the new mothers, involved themselves in social activities in their community. Thus, giving support was with the expectation that, the beneficiaries had or were expected to contribute in some way to their social network. Contribution to the network could be in different forms including adhering to social norms, respecting elders and providing support to others when needed. Thus, new parents, particularly mothers, who were considered to demonstrate poor social interactions, non-adherence to social norms and lack of respect for elders risked receiving less or no support from their informal social network as described in the following quote:

(...) if you live in harmony with other tenants [mmh] I do not think it will be impossible [to get support], but if you live in isolation, no one will take care of you.

FGD 11 Female supporters

Therefore, it was important for the new parents to be recognised as part of the informal social network to be able to expect support in future.

### 3. Informal support as gatekeeper to other forms of support

The informal supporters recognised the need for and regulated contact between the new parents and other sources of support. They indicated which situations or problems different stakeholders such as the health care system, traditional healers, governmental street and religious leaders and the police, could assist with, as expressed by one of the mothers below:

If you are staying with an adult person taking care of you for instance, you will try to tell her [about your concern], and if she tells you that it is not normal, then you are supposed to go to the hospital (...). You will see an expert and tell him your problem (...). If it is a common thing for mothers who have delivered, then they [support persons] will tell you so.

FGD 6 Mothers

The health care system was depicted as trusted and holding reliable knowledge and expertise in relation to certain health problems after childbirth, particularly infants' health problems. When home remedies, such as herbs, failed to work, formal health care services were the preferred authority. Informal supporters were described advising the new parents to consult health care facilities when facing health problems they themselves felt incompetent in solving.

I have to tell them [new parents] to seek advice from the doctor in the hospital where they gave birth. You have to go to the doctor, who shall know the problem facing the child. We have to go with time. In the present times, one cannot decide basing on the old beliefs. I might be afraid also because I do not have the instruments for investigations (...). May be the child's stomach is not in good shape! Therefore, it is the doctor's advice and not anyone else.

FGD 1 Male supporters

Despite many complaints about health workers' minimal involvement, their knowledge was considered trustworthy. Thus, more involvement from the health care system was expected during the period after childbirth. The services expected were identified as advice, treatment and care of ill infants, and health education on topics such as childcare, feeding, hygiene and crying. Information on sexuality, timing of sexual resumption and contraceptive use after childbirth were considered a part of the services required from the health care sector. Nevertheless, health workers were depicted as using both medical and traditional constructs when dealing with health matters after childbirth. The medical construct emphasizes the importance of exclusive breastfeeding for six months and encouraged the use of modern contraceptives. However, the traditional construct encouraged prolonged sexual abstinence in order to avoid child ill health in informal Swahili recognised as 'kubemenda'.

Though traditional healers were positioned as less trusted, the new parents were often advised to consult them for remedies or herbs for promoting quick and smooth recovery of the cord stump, prevent excessive crying, and prevent and cure 'kubemenda'. Traditional healers were depicted as useful when facing health problems associated with evil eyes, bad spirits or witchcraft. The main reason given for the mistrust of traditional healers was the presumed lack of expertise that led to misdiagnosis and mistreatment of their clients, especially infants as expressed here:

They [traditional healers] have their skills, but still you cannot trust on their skills hundred percent. You cannot look on one side, you must look on both sides (...). So when you give 'fungo' [herbs believed to protect a child], you must go to a health centre, you must also see a health expert.

FGD 6 Mothers

Informal supporters occasionally guided new parents to other formal sources of support as religious, governmental street leaders and police officers. Typically, this was to help with marital conflicts when they could not be solved within the family. To avoid shame and stigma, marital conflicts related to sexuality, including violence such as rape and battering of mothers, should not be reported to the police. Furthermore, police were portrayed as sometimes harassing women who reported violence to the police, expecting the perpetrators to be judged by the law. Thus, the discourse on informal support had elements that both facilitated and hindered the involvement of other stakeholders, depending on their judgement of the problem and the consequences of involvement for the family.

The dominant discourse advocating trust in informal, often traditionally oriented support and guidance was challenged by less dominant discourses based on preference for guidance from modern, often medically oriented support. Young people were considered modernised, as opposed to elders, who were depicted as maintaining traditional practices. New parents were sometimes described as not listening to the advice of supporters. With modern knowledge and technology, young parents were said to have new alternatives such as more access to health care and availability of condoms and other contraceptives. Both traditional and medical knowledge were sometimes questioned or disregarded due to lack of trust. Uncertainty on what and who to trust in different health promotional discourses was displayed. Trust between the support providers and receivers was of utmost importance, and ensured the support given was received and perceived as useful and was appreciated.

Tensions between the different prevailing discourses challenged the new parents and deciding on health promoting practices involved negotiating between different discourses, and could involve a choice between trusting the experience of the informal supporters or the command of medical knowledge among health workers. However, proximity and authoritative positions within the families favoured the opinions of the informal supporters and their continuous presence ensured the advice and instructions were most often followed by new parents. The dependency on the informal supporters implied at times obligation to comply with the instructions on health related matters, even against the parents own wish and health workers' advice as illustrated in the following quotes:

Whenever someone [informal supporter] comes in, she wants to give my child drinking water. (...). The person would say 'It is not good for the child to stay without being given drinking water for a long time even though the doctors advise not to give [water] until 6 months, but drinking water is very important'. So, you may find yourself giving the child drinking water, light porridge.

FGD 3 Mothers

In the hospital, we are told not to use hot water for massage. However, my mother and my aunt had to come with hot water secretly (...). They took me secretly from the ward and I got massaged for some weeks (...). My aunt then said, 'cases like these are not for hospital management'.

FGD 4 Female supporters

### 4. Upholding stereotypic norms of femininity and masculinity

The discourse embraced stereotypic norms of femininity and masculinity both on the 'recipient' and the 'provider' sides. New mothers were positioned as ignorant in matters related to their area of responsibilities such as care and health after childbirth. Experienced mothers were positioned as the ideal support people for the new mothers. New mothers were described as family carers in need of help, support and control, and new fathers were portrayed as financial providers, final decision makers and heads of the families, with less need for control from informal supporters. Only rarely were fathers described as providing a helping hand with infant care and household chores, and those who did, were sometimes depicted as weak men: some fathers were considered to use work as an excuse for their absence at home and supporting their partners. Physically, the home was the woman's place, and men were delineated as busy with paid employment outside the home.

Lack of economic means, made it difficult for mothers to fulfil social expectations of femininity and motherhood, and they were portrayed as facing increased difficulties in caring for themselves and their infants. The discourse was often in favour of a modern way of life, in which both men and women needed to have paid work and contribute financially to the family. However, the fathers' position in families was challenged if they were unable to provide financially for their families and failed to meet the prevailing expectations of fatherhood and masculinity. In such situations, women were described taking on paid work outside the home shortly after childbirth to support the family financially: this further undermined the partners' masculinity. The discourses on informal support i.e. control of the new mothers' life in general, and childcare and sexuality in particular, had no correspondence in the lives of the new fathers. Furthermore, women's agency in challenging gendered norms of female submissiveness and male dominance and decision-making power at home were not well received. References to religious books were made to ascertain gender relations as God-given orders that are unquestionable and cannot be challenged or changed by human beings.

You know women have perceived politics in a wrong way that we are equal. They do not differentiate equality in politics and in the home. Because even the Quran and the Bible clearly comment that 'The man is the head of the house'. But now equality issues are looked upon in wrong ways and forget those statements (...). This is what destroys many marriages (...). We have to agree that the man must have a final say and not a woman, in order to control the home. Because even the nation has a leader and the two cannot become leaders at the same time.

FGD 9 Male supporters

## Discussion

This study with first-time parents and informal support persons explored discourses on health-related informal support to first-time parents after childbirth in low-income, suburbs of Dar es Salaam, Tanzania. The subjective positioning of different actors during the period after childbirth was identified, and gender- power relations and health implications among families are presented. The discourses described in this study are based in the taken-for-granted understanding of the stakeholders in Ilala. They way people understand reality influences their social actions [40]. Consequently, unmasking existing discourses and opening them for discussion could contribute to change towards more health-promoting ways of understanding and acting. In this paper, formal support refers to support received from health care professionals while informal support refers to support received from non- health professionals within the community like family members, relatives, neighbours, friends and traditional healers.

Strong informal social networks were described as prevailing in traditional or reconstructed forms to fit the available sources of support in the study context, despite the risks of disruption of social ties that often follows internal migration [31,32]. The informal networks were constructed as providing practical help, information and guidance, and to some extent they helped the new parents to cope with socio economic difficulties. The reconstruction of traditional informal networks in the absence of close family members within the suburb was enabled by friends and other people in the neighbourhood. These findings demonstrate how important the social networks are in contributing to family health after childbirth, especially in the study context where health care system provides little attention over the period. This has practice implications on the health care provision and suggests a need to create linkage between the health care system and communities. The social networks could be used to complement the health care system if they provide appropriate messages and practice guidance to families.

However, support provision was delineated as being conditional, in that new parents should be involved in supporting others before they could be assured of receiving support themselves. Reciprocity, an aspect of social capital, is often important in social networks [21]. As a result, new parents were expected to adhere to the informal supporters' advice and guidance even when they did not agree. The discourse also revealed those who were poor often received less support. Thus, new parents who were migrant, poor and young often had less developed social networks and were likely to receive inadequate informal support from the surrounding community. This means the attention of the public health and social systems should be on providing support to those most in need, in order to maintain and promote family health after childbirth.

As with new parents in Western countries [18,42], the parents in this study appreciated the informal supporters' presence and the availability of help and guidance for promoting health in this new and stressful life situation. The informal support provided aspects of all four components of social support, as described by Bogossian [19] and has the potential to influence family health positively. However, not all advice and procedures promote health, despite the good intention of the supporters. Hot water vaginal or perineal compressions, prolonged sexual abstinence, infant mixed feeding, temporal confinement and separation are practices that could be questioned and discouraged, and alternatives based on medical knowledge suggested. Practices and norms that are harmless or likely to promote health such as provision of informal support in households, maternal resting and good nutritional practices should be encouraged.

Trust in the health care system, and expectations of more support from this system, were prominent in the discourse. The mediating role of the informal supporters needs to be appreciated and tapped for improving care and promoting family health after childbirth. The informal support gate keeping role could be utilized as a source of referrals connecting families in their networks to the health care system. The provision and accessibility of quality postpartum health care services, especially outreach programs, has the potential of being positively met by parents and informal supporters. However, there is a need to emphasise health workers training as some were described as recommending practices that did not promote health, such as mixed infant feeding and prolonged sexual abstinence.

Traditional healers were delineated as consulted to help solve some health and family matters despite doubts in their abilities and having access to health services in this context. This could be problematic, as despite being knowledgeable about social and cultural problems, the traditional healers were not always seen as good at preventing or curing other health problems. Neonatal infections resulting from traditional practices on newborns umbilical cord care are reported in Tanzania [43]. Traditional healers may be more important in contexts where health care facilities are not available, but irrespective of context, collaboration among traditional healers, health care providers and informal supporters in the community should be encouraged. The promotion of family health after childbirth should utilise all available support sources, and the first step towards this is to collaborate, and raise awareness and respect for different stakeholders' competencies and limitations.

Fist-time fathers were not positioned as recipients of informal support, apart from some aspects of sexuality and finance. Men's concerns while coping with first-time fatherhood appear to be overlooked in this Tanzanian setting [17], as in many Western countries [44-46]. Fathers' difficulties in coping with the new baby, expanded family responsibilities, and their partners' emotional and physical changes might affect the fathers' psychosocial wellbeing. Furthermore, fathers with partners who suffer depressive symptoms are at risk of developing depressive symptoms [44]. In Dar es Salaam, there is a 40% prevalence of depressive symptoms among pregnant and postpartum women [10], implying that many men may be at risk of developing the symptoms. In that context, fathers would also benefit from health interventions in the period after childbirth, both as fathers and partners.

The fathers were portrayed as rarely providing a helping hand with household chores and infant care, which was contrary to previous findings in the same setting [12,13,17]. The use of different data collection methods such as FGD and in-depth interviews [12,17] could partly explain this difference. In the FGD, people are likely to express perceptions or views that are socially acceptable as norms, whereas, in individual interviews personal perceptions are more often conveyed. None of the methods can reveal what is actually practiced, and the tendency to provide socially expected norms could apply to in-depth interviews. Interviews with first-time fathers [17] and mothers [12,13] are congruent, implying some validity to the findings. Thus, the reproduction of feminine and masculine roles in the discourse also played a role in the informants' understanding of the gender role divisions. The informal supporters were mainly women who coached the female parents to undertake their expected role of infant and family carers, implying the burden of family care was largely placed on the women, both older and younger mothers, and was congruent to previous findings [47]. Due to poor socioeconomic conditions, some women needed to work to contribute towards family finances, however, combining family care and work might be challenging if the women have to fulfil both expectations [12-14,47]. Moreover, fathers were described as financial providers and the final decision makers, as previously described in Tanzania [48,49]. However, the social positions of men with poor socioeconomic status could be threatened if they failed to meet the social expectations as family financial providers [49]: this conformed to the concept of multiple masculinities, where not all men fit into the dominant masculinity ideals worldwide [50]. However, men's social position as providers and final decision makers could be positively utilised as a resource for promoting family health, if they are involved in RCH matters. Raising men's awareness on health matters in relation to the period after childbirth would enlighten their understanding and enable them to make informed choices to promote family health. Furthermore, outreaches from existing health care programs could utilize experienced fathers as a source of guidance to new fathers by providing birth and complications preparedness messages. Even so, with the method used in the present study, we cannot claim the current findings represented the typical Tanzanian men and women's perceptions, a limitation for this study. Nonetheless, one can still conclude that gender stereotypes cannot be generalised.

If new parents, support people, traditional healers and health care providers received and delivered the same messages, it would be possible to diminish the stress caused by contradictory messages, as indicated in the discourses. Community and home based interventions in Nepal and Bangladesh [51,52] have indicated promising results in improving maternal and neonatal health. For example, in the Bangladesh study, community health workers made home visits to promote birth and newborn-care preparedness, made postnatal visits and identified and referred or treated sick newborns that resulted in reduced neonatal mortality. The study demonstrates how effective these low-cost interventions strategy could be used to promote health especially in communities with weak health-care systems as it is mostly the case in Tanzania context. Testing the feasibility of such interventions in a context specific forum for stakeholders at community and health facility levels could be the next step in preparing for an improved health-promoting environment for parents after childbirth.

## Conclusions

Strong and authoritative informal support networks appear to persist in the Ilala suburbs. However, poverty and non-adherence to social norms is understood as resulting in less support. Capitalising on existing informal support networks while discouraging norms promoting harmful practices and giving attention to the poorest is likely to improve family health in this context. Upholding stereotypic notions of femininity and masculinity implies great burden for the women in terms of care and delimits male involvement. However, men's involvement in reproductive and child health programmes has the potential for improving family health after childbirth. Furthermore, the prevailing discourses convey contradicting messages that can cause worry and confusion for the new parents. Recognition, respect and awareness of the different actors' competencies and limitations have the potential for creating a health-promoting environment among families after childbirth. It is recommended that future studies focus on exploring the different sources of informal support in urban areas, and norms, attitudes and practices and their prevalence in the period following childbirth from different ethnic groups. Furthermore, it would be interesting to study social, economic and ethnic variations among the population in relation to accessing social support within this complex social setting. Depressive symptoms that are most prevalent among postpartum women need to be explored among men.

## Competing interests

The authors declare that they have no competing interests.

## Authors' contributions

CKM has been involved in the conceptualization and designing of the study, field work coordination, data collection, data analysis and interpretation, and manuscript drafting; ABP was involved in conceptualization and designing of the study, data collection, data interpretation and critically revising the manuscript. KC and ED were involved in conceptualization and designing of the study and critically revising the manuscript. PO was involved in the conceptualization and designing of the study, data analysis and interpretation, critically revising the manuscript. All authors have read and approved the final manuscript.

## Pre-publication history

The pre-publication history for this paper can be accessed here:

http://www.biomedcentral.com/1471-2393/11/98/prepub
